# Supplementary material for: Characterization of Homeodomain Proteins at the Aβ Sublocus in Schizophyllum commune and Their Role in Sexual Compatibility and Development
Source: J Fungi (Basel). 2025 Jun 13;11(6):451. doi: 10.3390/jof11060451 (PMC12193891; doi:10.3390/jof11060451)
Supplement: Supplementary file 1 [file jof-11-00451-s001.zip › jof-3622649-supplementary.pdf]

Supplementary Material

Characterization of Homeodomain Proteins at the Aβ Sublocus in *Schizophyllum commune* and Their Role in Sexual Compatibility and Development

Chen Chu, Dongxu Li, Changhong Liu *

*** Correspondence:** Changhong Liu: chliu@nju.edu.cn

1 Supplementary Figures and Tables

1.1 Supplementary Figures

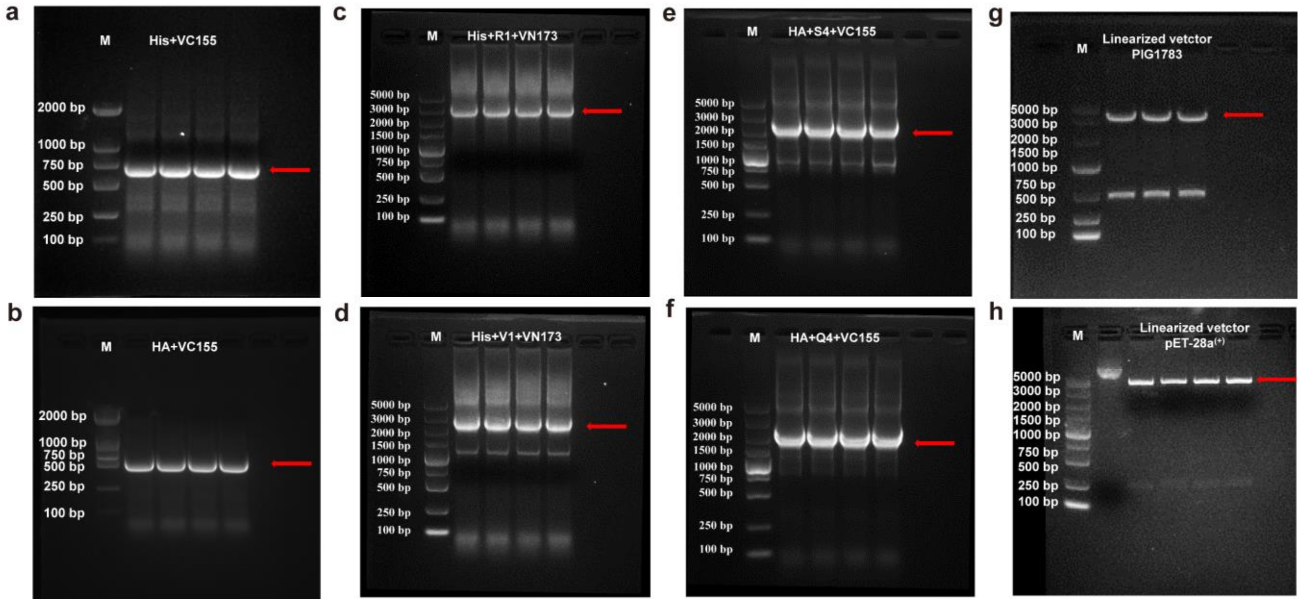

Figure S1. Construction of BiFC and protein expression plasmids. (a) His+VN173 fragment; (b) HA+VN173 fragment; (c) His+R1+VN173 fragment; (d) His+V1+VN173 fragment; (e) HA+S4+VC155 fragment; (f) HA+Q4+VC155 fragment; (g) Linearized pIG1783 vector used for *in vivo* BiFC plasmid construction; (h) Linearized pET-28a$^{(+)}$ vector used for *in vitro* protein expression plasmid construction.

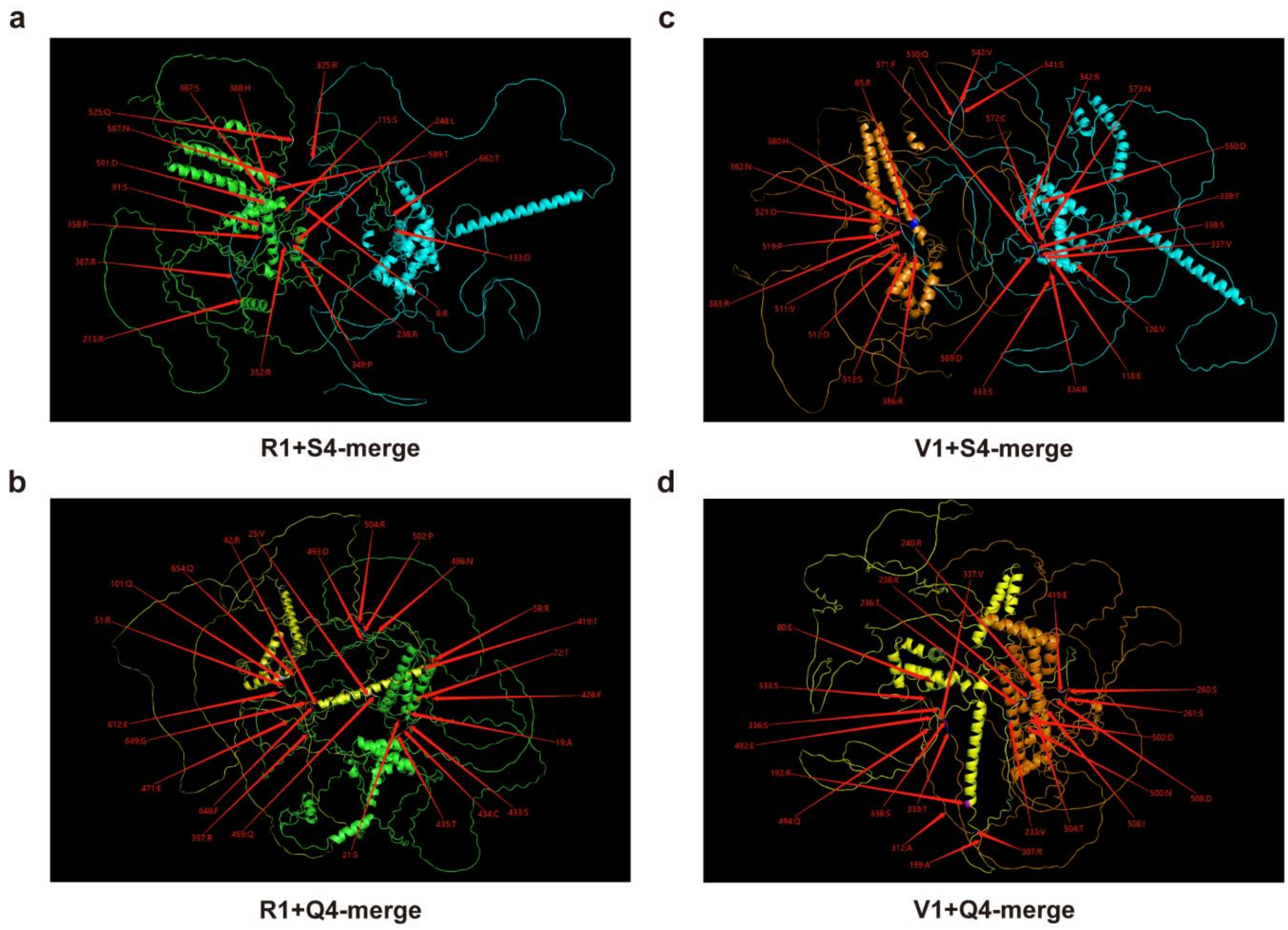

Figure S2. Protein interaction binding site annotation. Prediction binding sites for the interaction between (a) R1 and S4; (b) R1 and Q4; (c) V1 and S4; (d) V1 and Q4 proteins. Interaction binding sites were analyzed using PDBePISA and information was labeled by PyMOL.

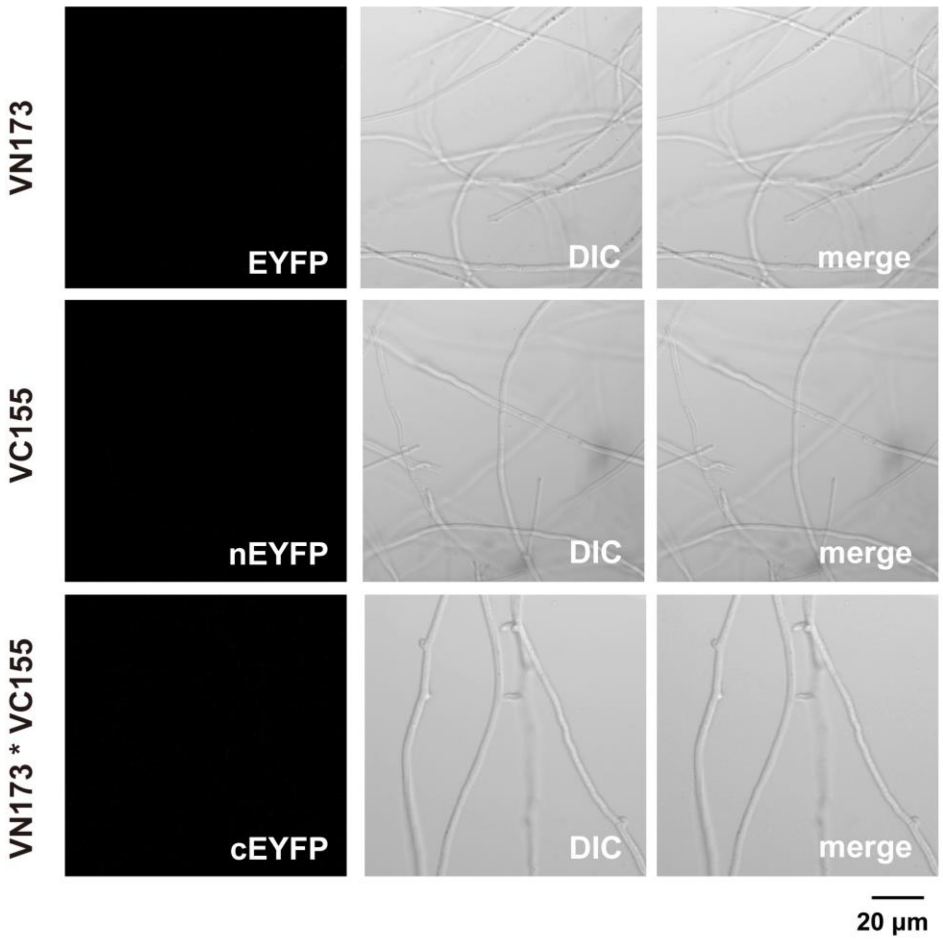

Figure S3. Empty vectors for the BiFC system. BiFC fluorescence images showing *in vivo* interaction between VN173 and VC155. Bar = 20 μm.

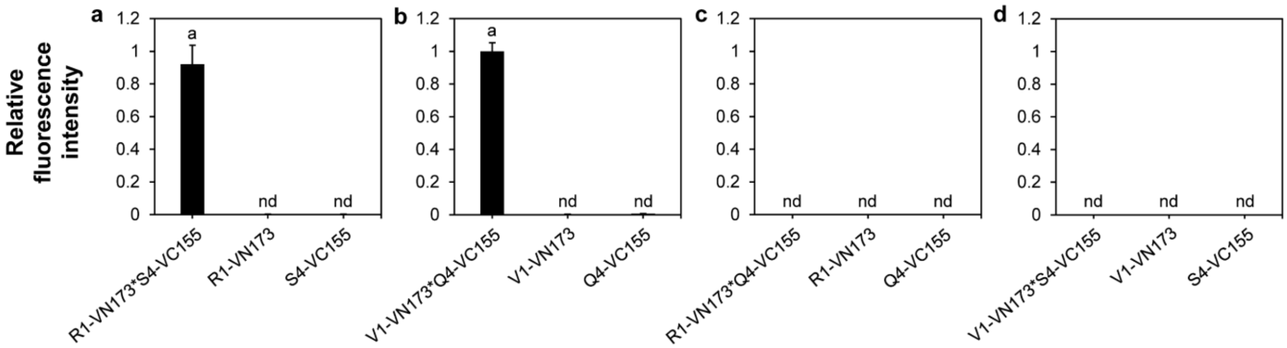

Figure S4. Relative fluorescence intensity of Figure 3 in the images. Values are presented as mean ± S.E (n = 20). Different letters indicate statistically significant differences ($p \leqslant 0.05$). nd = No fluorescence intensity detected.

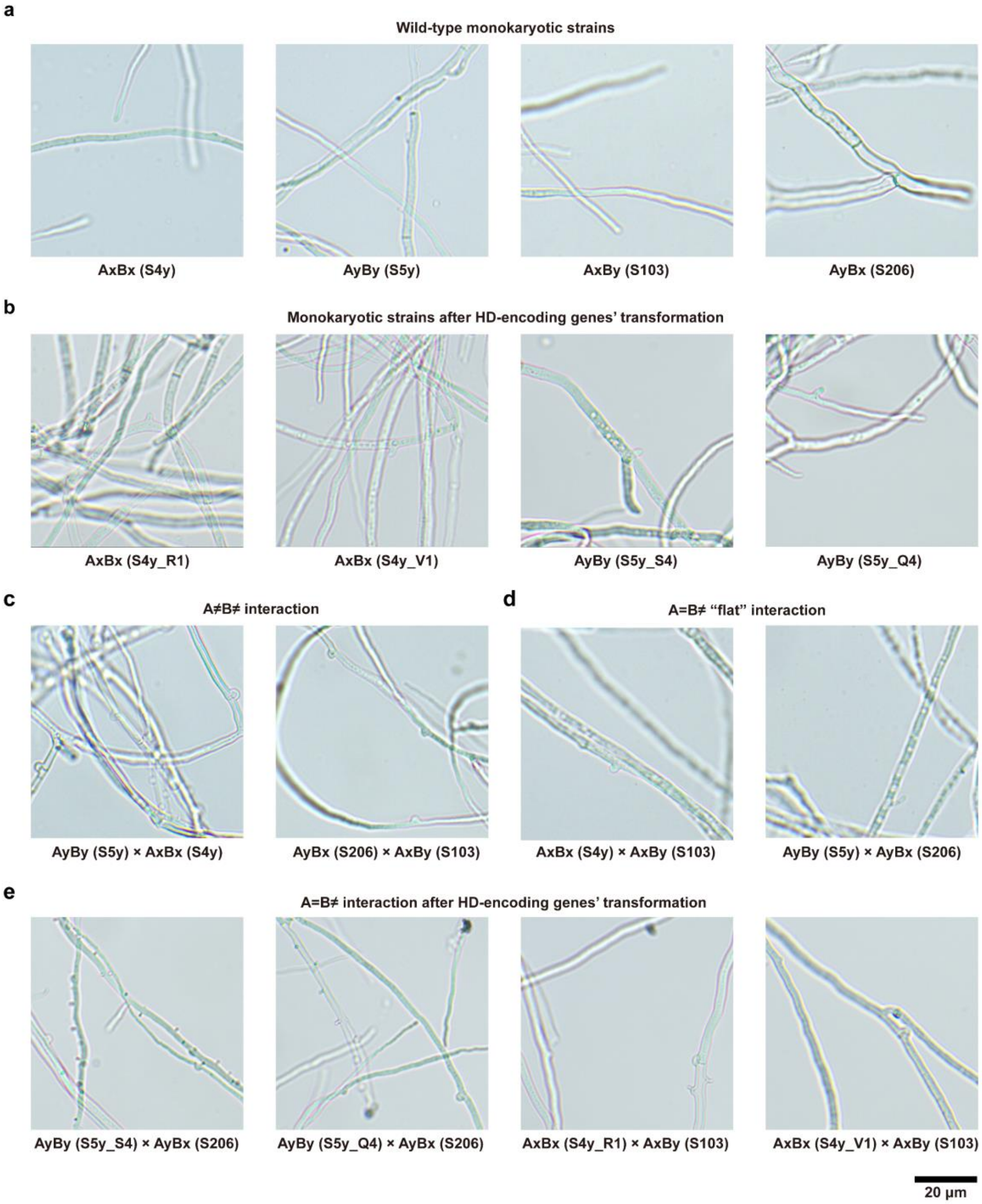

Figure S5. Microscopic observations of *S. commune* mycelium. Microscopic images of (a) wild-type monokaryotic strains; (b) monokaryotic strains after HD-encoding genes' transformation; (c) A≠B≠ interaction mycelium; (d) A=B≠ "flat" interaction mycelium and (e) A=B≠ interaction mycelium after HD-encoding genes' transformation. The strains representing each mating subtype are indicated in parentheses. Bar = 20 μm.

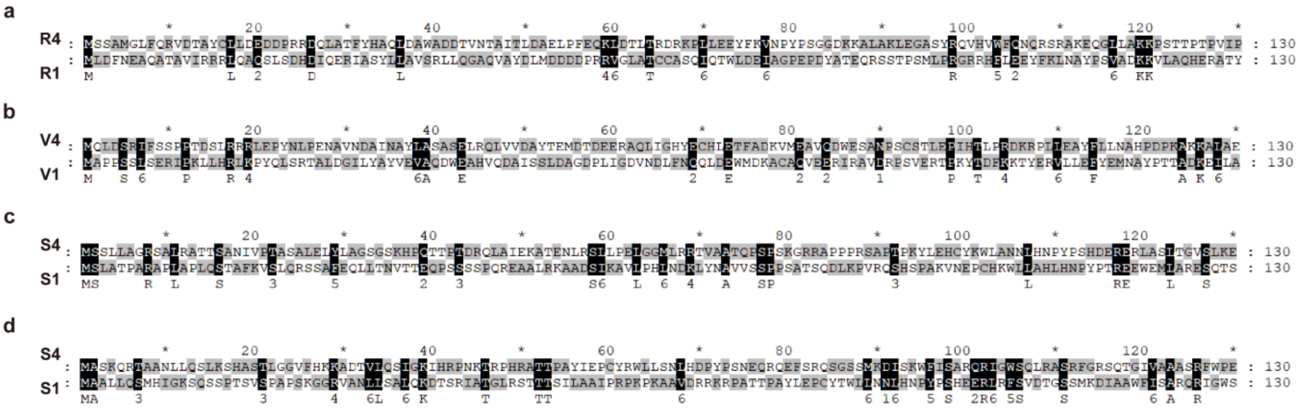

Figure S6. Multiple sequence alignment of R, S, V and Q proteins (amino acids 1-130). Multiple sequence alignment between (a) R4 and R1; (b) V4 and V1; (c) S4 and S1; (d) Q4 and Q1 proteins. Black and white coloring represents sequence homology, the higher the homology, the darker the coloring.

1.2 Supplementary Tables

Table S1. The *S. commune* strains used in this study.

Strains	Genotype	Description	Reference
20R-7-ZF01	Wild type (dikaryon)	Monokaryotic parent	Liu et al. (2017)
20R-7-F01-S4y	Wild type (monokaryon)	Protoplasmic preparation strain for transformation	This study
20R-7-F01-S4y_S4	pIG1783_S4-VC155	BiFC strain for C-terminal tagging of genes with VC155 (aa 520–772)	This study
20R-7-F01-S4y_Q4	pIG1783_Q4-VC155	BiFC strain for C-terminal tagging of genes with VC155 (aa 520–772)	This study
20R-7-F01-S4y_R1	pIG1783_R1	Functional validation strains for transformation of foreign interaction genes	This study
20R-7-F01-S4y_V1	pIG1783_V1	Functional validation strains for transformation of foreign interaction genes	This study
20R-7-F01-S5y	Wild type (monokaryon)	Protoplasmic preparation strain for transformation	This study
20R-7-F01-S5y_R1	pIG1783_R1-VN173	BiFC strain for N-terminal tagging of genes with VN173 (aa 1–519)	This study
20R-7-F01-S5y_V1	pIG1783_V1-VN173	BiFC strain for N-terminal tagging of genes with VN173 (aa 1–519)	This study
20R-7-F01-S5y_S4	pIG1783_S4	Functional validation strains for transformation of foreign interaction genes	This study
20R-7-F01-S5y_Q4	pIG1783_Q4	Functional validation strains for transformation of foreign interaction genes	This study
20R-7-F01-S103	Wild type (monokaryon)	Next-generation sequenced monokaryotic strain	This study
20R-7-F01-S206	Wild type (monokaryon)	Next-generation sequenced monokaryotic strain	This study

20R-7-F01-S17	Wild type (monokaryon)	Next-generation sequenced monokaryotic strain	This study
20R-7-F01-S192	Wild type (monokaryon)	Next-generation sequenced monokaryotic strain	This study
20R-7-F01-S180	Wild type (monokaryon)	Next-generation sequenced monokaryotic strain	This study
20R-7-F01-S10	Wild type (monokaryon)	Next-generation sequenced monokaryotic strain	This study

Table S2. Plasmids used in this study.

Plasmid	Genotype	Description	Source
pIG1783	—	Backbone vector for generation of BiFC plasmids	Pöggeler et al. (2003)
pET-28a(+)	—	Backbone vector for generation of protein expression plasmids	EMD Millipore
pIG1783-VN173	His-VN173 (1-519)	BiFC empty vector	This study
pIG1783-VC155	HA-VC155 (520-772)	BiFC empty vector	This study
pIG1783-R1-VN173	His-R1-VN173 (1-519)	BiFC plasmid for N-terminal tagging of genes with VN173 (aa 1–519)	This study
pIG1783-V1-VN173	His-V1-VN173 (1-519)	BiFC plasmid for N-terminal tagging of genes with VN173 (aa 1–519)	This study
pIG1783-S4-VC155	HA-S4-VC155 (520-772)	BiFC plasmid for C-terminal tagging of genes with VC155 (aa 520–772)	This study
pIG1783-Q4-VC155	HA-Q4-VC155 (520-772)	BiFC plasmid for C-terminal tagging of genes with VC155 (aa 520–772)	This study
pET-28a$^{(+)}$-His-R1	His-R1-VN173	Bait plasmid for His pull-down assay	This study
pET-28a$^{(+)}$-His-V1	His-V1-VN173	Bait plasmid for His pull-down assay	This study
pET-28a(+)-HA-S4	HA-S4-VC155	Prey plasmid for His pull-down assay	This study
pET-28a(+)-HA-Q4	HA-Q4-VC155	Prey plasmid for His pull-down assay	This study

Table S3. Primer sequences used.

Gene	Primer	Sequence (5'-3')	Product size (bp)
His label-VN173	PIG+His-F	TTGACTAACAGCTACCCCGCTTGAGCAGACATCACCatggATGGGCAGCAGCCATCATCATC	126
	VN173+His-R	agctcctcgcccttgctcaccatggcATGGCTGCCGCGCGGCACCAGGC	
VN173-HA	His+VN173-F	TGGTGCCGCGCGGCAGCCATgccatggtgagcaagggcgaggagc	585
	PIG+VN173-R	agtAACGTTAAGTGGATCCACTAGTTCTAGAGCGGCCGCTctactcgatgttgtggcggatcttg	
His+VN173-pIG1783	PIG+His-F	TTGACTAACAGCTACCCCGCTTGAGCAGACATCACCatggATGGGCAGCAGCCATCATCATC	665
	PIG+VN173-R	agtAACGTTAAGTGGATCCACTAGTTCTAGAGCGGCCGCTctactcgatgttgtggcggatcttg	
HA label-VC155	PIG+HA-F	TTGACTAACAGCTACCCCGCTTGAGCAGACATCACCatggatgtacccatacgatgttccagattacgc	201
	VC155+HA-R	accttgatgccgttcttctgcttgtcggcgtggttcatgactttctgtttc	
VC155-HA	HA+VC155-F	acctgaaacagaaagtcatgaaccacgccgacaagcagaagaacggcatc	320
	PIG+VC155-R	agtAACGTTAAGTGGATCCACTAGTTCTAGAGCGGCCGCTttacttgtacagctcgtccatgccg	
HA+VC155-pIG1783	PIG+HA-F	TTGACTAACAGCTACCCCGCTTGAGCAGACATCACCatggatgtacccatacgatgttccagattacgc	470
	PIG+VC155-R	agtAACGTTAAGTGGATCCACTAGTTCTAGAGCGGCCGCTttacttgtacagctcgtccatgccg	
His label-R1	PIG+His-F	TTGACTAACAGCTACCCCGCTTGAGCAGACATCACCatggATGGGCAGCAGCCATCATCATC	123
	R1+His-R	TGGGCCTCGTTGAAGTCGAGCATATGGCTGCCGCGCGGCACCAG	
abr1	His+R1-F	TCATCATCACAGCAGCGGCCTGGTGCCGCGCGGCAGCCATATGCTCGACTTCAACGAGGC	2364
	VN173+R1-R	tcctcgcccttgctcaccatggcGCCAGCAATCCCGAAGGTGAAG	
VN173-R1	R1+VN173-F	TTCACCTTCGGGATTGCTGGCgccatggtgagcaagggcgaggag	586

	PIG+VN173-R	agtAACGTTAAGTGGATCCACTAGTTCTAGAGCGGCCGCTctactcgatgttgtggcggatcttg	
His label-V1	PIG+His-F	TTGACTAACAGCTACCCCGCTTGAGCAGACATCACCatggATGGGCAGCAGCCATCATCATC	120
	V1+His-R	AATGAGGATGGTGGTGCCATATGGCTGCCGCGCGGCACCAG	
abv1	His+V1-F	TCATCATCACAGCAGCGGCCTGGTGCCGCGCGGCAGCCATATGGCACCACCATCCTCATTG	1971
	VN173+V1-R	tcctcgcccttgctcaccatggcGGCTGCAATAAGCGACGGTCCG	
VN173-V1	V1+VN173-F	TCGGACCGTCGCTTATTGCAGCCgccatggtgagcaagggcgaggag	588
	PIG+VN173-R	agtAACGTTAAGTGGATCCACTAGTTCTAGAGCGGCCGCTctactcgatgttgtggcggatcttg	
HA label-S4	PIG+HA-F	TTGACTAACAGCTACCCCGCTTGAGCAGACATCACCatggatgtacccatacgatgttccagattacgc	193
	S4+HA-R	TCCAGCGAGCAATGAGGACATgtggttcatgactttctgtttcag	
abs4	HA+S4-F	tgcaaaatcccgaacgacctgaaacagaaagtcatgaaccacATGTCCTCATTGCTCGCTGGAC	1952
	VC155+S4-R	accttgatgccgttcttctgcttgtcggcAGCAGCCGCAGGGGCGAGCC	
VC155-S4	S4+VC155-F	AGGCTCGCCCCTGCGGCTGCTgccgacaagcagaagaacggcatc	319
	PIG+VC155-R	agtAACGTTAAGTGGATCCACTAGTTCTAGAGCGGCCGCTttacttgtacagctcgtccatgccg	
HA label-Q4	PIG+HA-F	TTGACTAACAGCTACCCCGCTTGAGCAGACATCACCatggatgtacccatacgatgttccagattacgc	193
	Q4+HA-R	TGTGCGCTGCTTGGAGGCCATgtggttcatgactttctgtttcag	
abq4	HA+Q4-F	tgcaaaatcccgaacgacctgaaacagaaagtcatgaaccacATGGCCTCCAAGCAGCGCACAGC	1772
	VC155+Q4-R	accttgatgccgttcttctgcttgtcggcTGCCGTCGCAGCGATAGGCTC	
VC155-Q4	Q4+VC155-F	AGCCTATCGCTGCGACGGCAgccgacaagcagaagaacggcatc	318
	PIG+VC155-R	agtAACGTTAAGTGGATCCACTAGTTCTAGAGCGGCCGCTttacttgtacagctcgtccatgccg	

His+R1/V1+VN173-pIG1783	PIG+His-F	TTGACTAACAGCTACCCCGCTTGAGCAGACATCACCatggATGGGCAGCAGCCATCATCATC	R1-2966 / V1-2573
	PIG+VN173-R	agtAACGTTAAGTGGATCCACTAGTTCTAGAGCGGCCGCTctactcgatgttgtggcggatcttg	
HA+S4/Q4+VC155-pIG1783	PIG+HA-F	TTGACTAACAGCTACCCCGCTTGAGCAGACATCACCatggatgtacccatacgatgttccagattacgc	S4-2351 / Q4-2171
	PIG+VC155-R	agtAACGTTAAGTGGATCCACTAGTTCTAGAGCGGCCGCTttacttgtacagctcgtccatgccg	
His+R1/V1+VN173-pET-28a(+)	28a+His-F	ataccatgggcagcagccatcatcatcatcatcacagcagcggcctggtgccgc	R1-2918 / V1-2525
	28a+VN173-R	tgctgtccaccagtcatgctagccatactactcgatgttgtggcggatcttgaag	
HA+S4/Q4+VC155-pET-28a(+)	28a+HA-F	attttgtttaactttaagaaggagatatacatgtacccatacgatgttccagattacgc	S4-2333 / Q4-2153
	28a+VC155-R	tagaggccccaaggggttatgctagttattgcttacttgtacagctcgtccatgccgag	

Table S4. The *MatA* genes homology comparison by BLASTP.

Mating type	Gene Type		Genes	Blast_ID	Identity	Coverage	Evalue
**20R-7-F01_Ax**	**Homeodomain Genes**	**HD2**	*abr4*	XP_003037496.1	46.677	85	4.04E-138
			abv4	XP_003037495.1	42.143	28	8.03E-25
			aay4	XP_003038722.1	92.142	100	0
		**HD1**	*abs4*	XP_003038830.1	65.615	96	0
			abq4	XP_003037497.1	41.424	54	9.17E-56
			aaz4	XP_003037384.1	85.714	100	0
	**Mitochondrial Intermediate Peptidase Gene**	**MIP**	*mip*	P37932.2	98.71	100	0
	**Beta-fg Gene**	**Beta-fg**	*β-fg*	XP_003038552.1	98.624	100	1.82E-158
**20R-7-F01_Ay**	**Homeodomain Genes**	**HD2**	*abr1*	XP_003037496.1	59.41	100	0
			abv1	XP_003037495.1	31.56	44	6.41E-29
			aay1	AAB01367.1	90.556	99	0
		**HD1**	*abs1*	XP_003038830.1	43.729	50	1.87E-55
			abq1	XP_003037497.1	39.809	53	6.83E-54
	**Mitochondrial Intermediate Peptidase Gene**	**MIP**	*mip*	P37932.2	98.968	100	0
	**Beta-fg Gene**	**Beta-fg**	*β-fg*	XP_003038552.1	99.083	100	2.62E-159
